# The epigenetic immunomodulator, HBI-8000, enhances the response and reverses resistance to checkpoint inhibitors

**DOI:** 10.1186/s12885-021-08702-x

**Published:** 2021-08-30

**Authors:** Reid P. Bissonnette, Rosemary M. Cesario, Bob Goodenow, Farbod Shojaei, Mireille Gillings

**Affiliations:** HUYABIO International, LLC, San Diego, CA USA

**Keywords:** Histone deacetylase, HDAC, Epigenetics, PD-1, PD-L1, Immune checkpoint, Dendritic cells, Checkpoint blockade

## Abstract

**Background:**

Treatment with immune checkpoint inhibitors (ICIs) targeting CTLA-4 and the PD-1/PD-L1 axis is effective against many cancer types. However, due in part to unresponsiveness or acquired resistance, not all patients experience a durable response to ICIs. HBI-8000 is a novel, orally bioavailable class I selective histone deacetylase inhibitor that directly modifies antitumor activity by inducing apoptosis, cell cycle arrest, and resensitization to apoptotic stimuli in adult T cell lymphoma patients. We hypothesized that HBI-8000 functions as an epigenetic immunomodulator to reprogram the tumor microenvironment from immunologically cold (nonresponsive) to hot (responsive).

**Method:**

Mice bearing syngeneic tumors (MC38 and CT26 murine colon carcinoma and A20 B-cell lymphoma were treated daily with HBI-8000 (orally), alone or in combination with PD-1, PD-1 L, or CTLA-4 antibodies. MC38 tumors were also analyzed in nanoString gene expression analysis.

**Results:**

HBI-8000 augmented the activity of ICI antibodies targeting either PD-1, PD-L1 or CTLA-4, and significantly increased tumor regression (*p* < 0.05) in the above models. Gene expression analysis of the treated MC38 tumors revealed significant changes in mRNA expression of immune checkpoints, with enhanced dendritic cell and antigen-presenting cell functions, and modulation of MHC class I and II molecules.

**Conclusions:**

These findings suggest that HBI-8000 mediates epigenetic modifications in the tumor microenvironment, leading to improved efficacy of ICIs, and provide strong rationale for combination therapies with ICIs and HBI-8000 in the clinical setting.

**Precis:**

As an HDACi, HBI-8000 plays an important role in priming the immune system in the tumor microenvironment. The current preclinical data further justifies testing combination of HBI-8000 and ICIs in the clinic.

**Supplementary Information:**

The online version contains supplementary material available at 10.1186/s12885-021-08702-x.

## Background

Advances in cancer immunotherapy, starting with the approval of immunotherapeutic agents targeting cytotoxic T-lymphocyte-associated protein 4 (CTLA-4), the programmed cell death receptor-1 (PD-1), and the PD-1 ligand (PD-L1), have drastically improved the treatment of a wide range of cancer types, including difficult-to-treat solid tumor cancers. Durable clinical responses, however, occur in only 10 to 45% of patients, and remaining patients are either innately unresponsive or develop resistance and relapse [1–3]. On this basis, researchers have sought to identify modalities with potential additive or synergistic effects when combined with immunotherapies. Efforts to understand the mechanisms of immune checkpoint inhibitor (ICI) nonresponsiveness or resistance have revealed the importance of epigenetic changes in both tumor and immune cells within the tumor microenvironment (TME) and the potential to manipulate several facets of antitumor immunity with epigenetic immunomodulators [4–6]. The reversal of ICI nonresponsiveness/resistance may include the re-expression of silenced or dysregulated genes that modulate immune recognition and elimination of tumor cells and overcoming an immunosuppressive tumor or systemic environment [4, 7–10]. The mechanism of action of ICIs may not be driving the reinvigoration of pre-existing effector T cells with an exhausted phenotype, but rather supporting the generation of novel tumor-selective T cell clones [11]. Thus, the clinical activity of ICIs requires effective presentation of tumor antigens to T and B cells, highlighting the role of antigen-presenting cells, dendritic cells, the expression of major histocompatibility complex (MHC) class I and class II molecules, and the resulting de novo generation of tumor-selective T and B cell clones. Not surprisingly, numerous reports point to the dysregulation of antigen presentation machinery and loss of MHC and β-2 microglobulin expression as important mechanisms of tumor resistance to ICI therapy [12–15].

Class I-selective histone deacetylase inhibitors (HDACi) enhance antitumor immune responses in multiple preclinical models through epigenetic modifications, including histone hyperacetylation and DNA demethylation events. These changes in the tumor epigenome can reverse clinical drug resistance and mediate a return to treatment sensitivity [16]. HBI-8000 is a clinically validated, orally bioavailable class I- (HDAC1, 2, and 3) selective HDACi. HBI-8000 has direct anti-tumor capacity in adult T cell lymphoma patients via the induction of cell cycle arrest and apoptosis. In addition to directly targeting cancer cells, HBI-8000 has positive effects on antitumor immunity, enhancing the activity of both cytotoxic T lymphocytes and natural killer (NK) cells [17–32]. The original observations by West et al. [33, 34] that the anticancer effects of HDACi are dependent on an intact immune system have encouraged several laboratories to investigate the effects of drugs that affect epigenetic changes on antitumor immunity. Recent reports suggest that HDACi has a significant effect on the expression of immune checkpoint co-inhibitory and co-stimulatory molecules. Additionally, HDACi may affect immunogenicity, antigen-presenting cell and T cell priming, regulatory T cells, myeloid-derived suppressor cells, and effector cell functions [16, 35–38].

Investigation on the role of epigenetics in response to ICIs, together with the need to evaluate rationale immunotherapy combinations, led us to hypothesize that HBI-8000 functions as an epigenetic immunomodulator to reprogram the TME, converting immunologically cold (nonresponsive) tumors to hot (responsive) tumors. To test this hypothesis, we combined HBI-8000 with several ICIs, i.e., antibodies targeting PD-1 (PD-1 Ab), PD-L1 (PD-L1 Ab), and CTLA-4 (CTLA-4 Ab) to treat tumors transplanted into several different immune-competent mouse models. Our findings revealed that HBI-8000 enhanced the antitumor activity of all 3 ICIs tested, as reflected by increased inhibition of tumor growth in several preclinical models. To better understand the mechanism of this enhancement, we employed the NanoString nCounter PanCancer Immune Profiling panel to evaluate the gene expression profile in components of the immune system in the TME. Clustering analysis revealed that HBI-8000 mediated changes in immune response-relevant genes co-clustered with those induced by the combination of HBI-8000 plus PD-1 Ab, suggesting that HBI-8000 primes (induces the activity of components of tumor immunity against cancer cells) the TME [39, 40]. HBI-8000 modulated the expression of several immune checkpoints and immune response-relevant genes, all of which associated with an effective antitumor response, suggesting a role for HBI-8000 in converting the TME from cold to hot [41–44]. Finally, in a model in which the initial response to ICI therapy often leads to tumor progression and resistance, the combination of HBI-8000 and ICI therapy delayed tumor growth in ~ 50% of mice progressing on PD-1 Ab therapy. These findings further elucidate the potential of epigenetic immunomodulators like HBI-8000 to enhance the activity of ICIs and serve as a rationale for the development of combination therapy for clinical application.

## Methods

### Cell lines and reagents

The MC38 and CT26 syngeneic murine colon carcinomas were obtained from ATCC (Manassas, VA), and the A20 cells were obtained from Covance (Princeton, NJ). Cells were passaged and maintained using the protocols provided by the vendors. HBI-8000 was supplied by HUYA Bioscience International. HBI-8000 (HUYA Bioscience International) was formulated in 10% hydroxypropyl- β-cyclodextrin and 10% propylene glycol in deionized water, pH 2.5. Dosing solutions were prepared fresh weekly and stored at 4 °C. Animals were dosed orally daily with 50 mg/kg HBI-8000 for 21 days.

Monoclonal antibodies (mAbs) to mouse PD-1 (clone RPM-14), PDL-1 (clone (10F.9G2), and CTLA-4 (clone 9H10) were purchased from Bio-X-Cell (West Lebanon, NH). Antibody dosing solutions were prepared in sterile phosphate-buffered saline on each dosing day, and stored at 4 °C. Mice were intraperitoneally injected with the PD-1 antibody (Ab) or PD-L1 Ab (10 mg/kg) twice weekly for 3 weeks. CTLA-4 Ab (2.5 mg/kg) was administered intraperitoneally on days 1, 4, and 7.

### Animal models and in vivo treatment

All animal research studies were approved and overseen by the Institutional Animal Care and Use Committees of Charles River (MC38, CT26). All mice obtained from Charles River (Morrisville, NC) were female and 8 weeks old when the tumors were implanted. For MC38 tumors, C57BL/6 mice were implanted subcutaneously in the right flank with 1 × 10^6^ MC38 cells (0.1-mL cell suspension). For CT26 tumors, BALB/c mice were injected subcutaneously in the right flank with 3 × 10^5^ CT26 tumor cells (0.1-mL cell suspension). For A20, BALB/c mice were implanted subcutaneously in the right flank with 1 × 10^6^ A20 cells (0.1-mL cell suspension). Tumor growth was monitored until reaching an average volume of 100 mm^3^, at which time (day 0) the mice were randomized into the various treatment groups. Treatments were initiated on day 1. Tumor volume was calculated using caliper measurements according to the following formula:

$$ \mathrm{Tumor}\ \mathrm{volume}\ \left({\mathrm{mm}}^3\right)=\frac{w^2\ x\ l}{2}, $$

where w = width and l = length (in mm) of the tumor.

### PD-1 failure and rescue studies

To establish a model of PD-1 antibody failure or stable disease, 150 mice were initially treated biweekly for 3 weeks with first-line anti-PD-1 Ab (5 mg/kg, intraperitoneal administration). Mice bearing tumors that exhibited either slow progression or stable disease was defined as 3 consecutive measurements with no significant change in tumor volume) were subsequently re-enrolled into second-line therapy groups (*n* = 10/group) including Vehicle, HBI-8000, PD-1 Ab, PD-1 Ab plus HBI-8000, PD-L1 Ab, and PD-L1 Ab plus HBI-8000.

### NanoString nCounter PanCancer immune profiling panel gene expression studies

Gene expression studies were carried out using excised MC38 tumors (*n* = 20 animals/treatment) isolated from syngeneic C57BL/6 mice treated for 7, 14, or 17 days with HBI-8000 (50 mg/kg, daily), anti-PD-1 (10 mg/kg, biweekly), or the combination of HBI-8000 + anti-PD-1 (50 mg/kg, daily, 10 mg/kg, biweekly). At study termination, tumor samples from the treated mice were collected and fixed in formalin for 24 h and transferred to EtOH, followed by the preparation of formalin-fixed paraffin embedded (FFPE) blocks. Tumor sections (5–10 μm) were prepared from the FFPE blocks, and total RNA was isolated from tissue scraped from 4 to 6 slides using the protocol recommended by NanoString Technologies (Seattle, WA). The nCounter PanCancer Immune Profiling panel developed and provided by NanoString Technologies was initially selected for expression analyses with an additional 20 genes added as a Panel Plus Codeset. The additional genes were predicted to be regulated by HBI-8000 +/− ICI treatment. The nCounter assays were performed according to the manufacturer’s instructions using the nCounter FLEX system.

Gene expression data were analyzed using nSolver software provided by NanoString Technologies, Inc. Raw data were normalized to the geometric mean values of the internal synthetic positive controls and geometric means of the housekeeping genes, as recommended by the manufacturer. The NanoString Technologies’ nSolver Analysis Software 4.0 generated cell type scores, pathway scores, heatmaps, and individual gene normalized data from the nCounter PanCancer Immune Profiling Panel Plus dataset. The cell type score quantifies cell populations using marker genes for given cell types; by centering the mean at 0 for each cell type, immune cell type abundance can be compared on the same scale. The same method was used to generate immune-relevant pathway scores; summarizing the data from multiple genes in a pathway into a single score allowed for comparison between treatments for pathway analysis.

Normalized expression data for individual genes was exported from nSolver, annotated with percent of tumor growth inhibition (%TGI), and then imported into GraphPad Prism 7.04. The %TGI was used to group animals into 3 categories, as follows: nonresponders (TGI < 25%), partial responders (TGI 25–75%), and responders (TGI > 75%). Gene expression data for each mouse was color-coded (TGI < 25%, TGI 25–75%, TGI > 75%) to track gene expression with tumor response and used to determine if changes in gene expression associated with the tumor response.

### Statistical analyses

Prism 7.04 (GraphPad, San Diego, CA) was employed for statistical and graphical analyses. Survival was analyzed by the Kaplan-Meier method. The logrank (Mantel-Cox) and Gehan-Breslow-Wilcoxon tests determined the significance of the difference between the overall survival experiences (survival curves) of two groups, based on time to endpoint values. Differences in tumor size among groups were assessed using 2-tailed t-test statistical analyses. The results are reported as nonsignificant (ns) at *P* > 0.05, significant (*) at 0.01 ≤ *P* < 0.05, very significant (**) at 0.001 ≤ *P* < 0.01, and extremely significant (***) at *P* < 0.001.

Studies were carried out in compliance with the ARRIVE guidelines.

## Results

### Combining HBI-8000 with antagonist mAbs to mouse PD-1, PD-L1, and CTLA-4 enhances the antitumor responses and leads to tumor regression

**Fig. 1:**
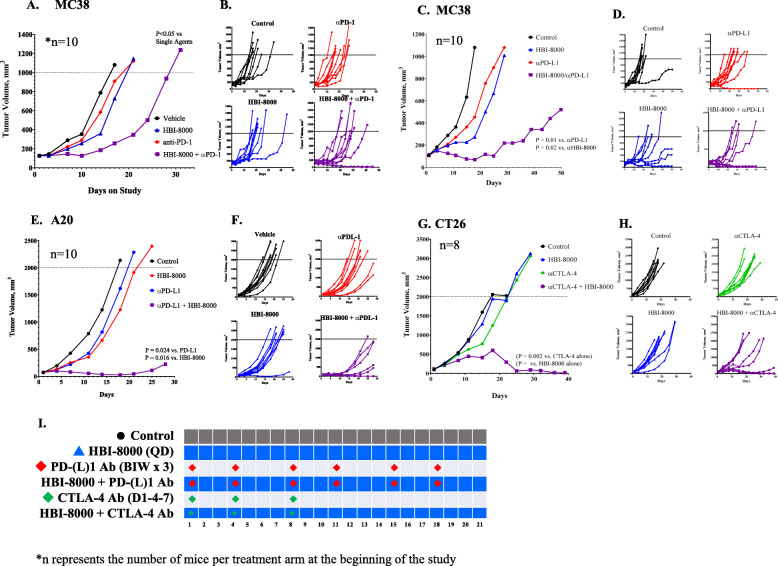
Tumor growth inhibition (TGI) in mice treated with ICI, HBI-8000, or their combination. Syngeneic MC38 (**A-D**), A20 (**E & F**) and CT26 (**G & H**) tumors were implanted in C57BL/6 or BALB/c mice and were allowed to grow until the mean tumor volume was ~ 100 mm^3^. Animals were then randomized into groups with equivalent mean tumor volumes and treated with the indicated therapeutic agents. Data shown represent the median tumor volume for each treatment group at the indicated day post-initiation of therapy (**A, C, E and G**), as well as the individual tumor volumes per animal (**B, D, F and H**). **I** Dosing regimens and intervals

To test whether HBI-8000 augments the antitumor effects of inhibiting the PD-(L)1 immune checkpoint axis, we treated mice bearing MC38 syngeneic tumors with HBI-8000, a mouse PD-1 Ab, or HBI-8000 plus PD-1 Ab (Fig. 1A, B). Therapeutic regimens and intervals are depicted in Fig. 1I. Figure 1B shows tumor growth in individual mice. In addition, we treated MC38 tumor-bearing mice with HBI-8000, a mouse PD-L1 Ab, or HBI-8000 plus PD-L1 Ab (Fig. 1C, D). Treatment with a single agent (HBI-8000, PD-1 Ab, or PD-L1 Ab) did not significantly affect tumor growth. Tumor regression (i.e., absence of detectable tumor) was not seen in any of the single agent cohorts, and all tumors continued to grow throughout the study. In contrast, combining either the PD-1 Ab or the PD-L1 Ab with HBI-8000 produced a statistically significant and reproducibly synergistic decrease or delay in tumor growth and progression (Fig. 1A, C). To corroborate these results, we extended our investigations to 2 other syngeneic tumor models. Mice bearing A20 tumors were treated with the same modalities and similar results were generated. Single-agent HBI-8000, PD-1 Ab, or PD-L1 Ab (A20, Fig. 1E, F) did not significantly affect tumor growth. As seen in the MC38 model, however, the combination of either a PD-1 Ab or PD-L1 Ab with HBI-8000 produced a significant and synergistic decrease or delay in tumor growth and progression, and importantly, an increase in the number of mice with tumor regression. Finally, we tested HBI-8000, a mouse CTLA-4 Ab, or HBI-8000 plus CTLA-4 Ab in the CT26 model (Fig. 1G, H). Similar to ICIs targeting PD-1 and PD-L1, the CTLA-4 Ab alone did not significantly affect tumor growth. Combining HBI-8000 with CTLA-4 Ab produced a highly significant delay in tumor progression, with 20% of tumor-bearing mice experiencing complete regression.

In summary, irrespective of the mouse tumor model or ICI Ab, single-agent therapy did not inhibit/regress tumor growth in any of the models tested. In all treatments combined with HBI-8000, we observed tumor regression after treatment, with subsets of tumors showing a significant delay in progression or outright regression. The data indicate that combining HDACi HBI-8000 with an ICI Ab was very efficacious in multiple animal models.

### HBI-8000 epigenetically reprograms the TME and increases the expression of genes indicating enhanced antigen presentation, dendritic cell function, and effector cell antitumor cytotoxicity

**Fig. 2:**
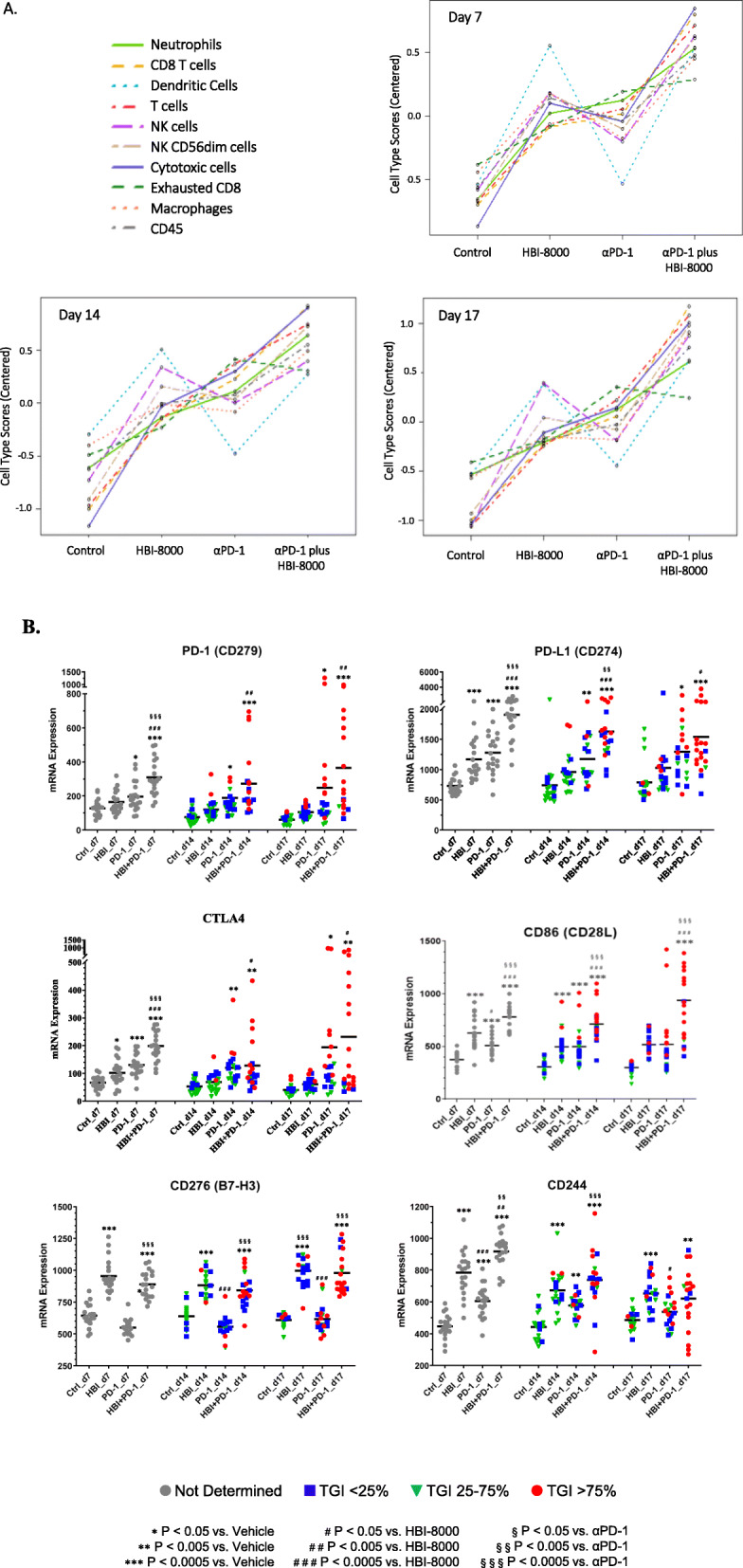
Immune cell-types and pathways modulated by PD-1 Ab, HBI-8000, or their combination. Syngeneic MC38 tumors were implanted in C57BL/6 mice and allowed to grow until the mean tumor volume was ~ 100 mm^3^. The mice were then randomized into groups of 20 mice with equivalent mean tumor volumes and treated with the indicated therapeutic agents. At days 7, 14, and 17, groups of 20 mice were killed, and the tumors were excised, fixed in formalin, and embedded in paraffin. Tumor sections were then processed for nCounter gene expression analysis as described in the Methods. **A**. Plots of the immune cell types in the TME modulated by PD-1 Ab, HBI-8000, or their combination at days 7, 14, and 17 for each treatment group. **B**. Immune checkpoints (PD1, PD-L1, CTLA4, CD86, CD276, and CD244) modulated by PD-1 Ab, HBI-8000, or their combination. The data depict the mRNA expression levels for each gene at days 7, 14, and 17. Statistical significance is as indicated in the graphs. Individual mice were tagged according to the antitumor response. Red circles () represent TGI > 75%, inverted green triangles () TGI from 25% through 75%, and blue squares () were assigned to mice with TGI < 25%

To investigate the mechanism of action of HBI-8000 in combination therapy with ICIs, larger groups of mice (*n* = 20/group) were implanted with MC38 tumors to sufficiently power the statistical analysis. A baseline no-treatment tumor-bearing group was terminated 1 day before initiating treatment. Mice in each treatment arm (*n* = 20) were terminated at days 7, 14, and 17 post treatment initiation. The NanoString nCounter PanCancer Immune Profiling Panel analysis allows for clustering of immune response-related genes into “gene sets” comprising a collection of genes selected as being representative of an element of the immune response (i.e., cell type, pathway), and provides a high-level view of the antitumor response, which is depicted as scatterplots in Fig. 2A. While all the scores were elevated in the PD-1 Ab plus HBI-8000 combination agent cohorts, it is noteworthy that a subset of cell type scores was augmented by either PD-1 Ab or HBI-8000 alone as early as day 7. Scores for exhausted CD8 T cells and neutrophils were predominantly augmented by the PD-1 Ab. In contrast, HBI-8000 augmented the scores corresponding to dendritic cells, macrophages, NK cells, cytotoxic cells, and CD45 cells, demonstrating that HBI-8000 alone had a profound conditioning or priming effect on immune-relevant gene expression within the TME, and suggesting that it reprograms the TME such that ICI therapy is more effective. Interestingly the same trends is observed in Day 14 and Day 17 analysis indicating consistency in HBI-8000 effects on TME as single agent or in combination with PD-1 antibody.

The de novo generation of new tumor-selective T cell clones might be a key factor in the response to the PD-1/PD-L1 checkpoint blockade [11, 36]. Because data from a preliminary study suggested that HBI-8000, alone or in combination with PD-1 Ab, has profound effects on the early or priming phase of the immune response, we investigated changes in the expression of genes associated with dendritic cell functions, antigen processing, and MHC class II antigen presentation. Consistently, gene expression analysis in single agent HBI-8000–treated tumors showed at least partial co-clustering with the response to the HBI-8000 plus ICI Ab combination therapy within these gene sets (Shown by yellow rectangle, Supplemental Figure 1A). HBI-8000 also co-clustered with combination therapy at the level of MHC class I antigen expression and presentation, which is important for effector T cell recognition and killing of tumor cells (Supplemental Figure 1B). Unsupervised hierarchical clustering of the indicated immune cell type scores vs. treatment and tumor response (Supplemental Figure 1A) showed that gene expression changes representative of these scores were most notable in the PD-1 Ab plus HBI-8000 combination cohort, and in responders vs. nonresponders (Supplemental Figure 1B). The analysis also demonstrated segregated clustering of the adaptive vs. innate response cells types. Not surprisingly, PD-1 Ab plus HBI-8000 combination therapy co-clustered with gene expression sets representing high response rates (TGI > 75%), which was observed for both adaptive and innate immune cell types. HBI-8000 also co-clustered with the HBI-8000/PD-1 Ab combination in modulating the expression of gene sets associated with cytokines, chemokines and their receptors, and with adaptive immunity-related genes. The data suggest that the class I/II selective HDACi can epigenetically modulate gene expression patterns within the TME, which contributes to multiple facets of the antitumor immune response, leading to the priming of effector T and B cells, the recognition of tumor cells by T cells with a consequent shift in the expression of relevant cytokines and corresponding receptors, and the augmentation of both innate and adaptive immune responses.

### HBI-8000 alone or in combination with PD-1 Ab induces changes in several immune checkpoints within the TME

Induction of immune checkpoint receptors and/or ligands is thought to indicate a shift from a T cell-noninflamed (cold) TME to a T cell-inflamed (hot) TME [45]. The changes observed in immune checkpoints in the MC38 TME are shown in Fig. 2B. The data plots are color-coded to represent the tumor growth inhibition response seen for each individual animal, set arbitrarily for the purpose of illustration as tumor growth inhibition > 75, 25% through 75%, or less than 25% to represent responders, stable disease, and progressors, respectively. We observed increased expression of the immune checkpoints PD-1, PD-L1, CTLA-4, and CD86 (CD28L), the expression levels of which associated with antitumor efficacy and tumor regression (Fig. 2B). We also observed statistically significant changes in the expression of immune checkpoints CD276/B7-H3 and CD244 (Fig. 2B), as well as lymphocyte activation gene-3 (LAG-3), T cell immunoreceptor with Ig and ITIM domains (TIGIT), ecto-5′-nucleotidase (NT5E/CD73), signal regulatory protein α (SIRPα), nuclear factor of activated T cells 4 (NFATC4), and poliovirus receptor (CD155; Supplemental Figure 2). We believe modulation in the expression of the above genes indicates a shift from a noninflamed (cold) TME to an inflamed (hot) TME and interrelated with the antitumor response in MC38 tumor-bearing mice [42, 45–47].

### HBI-8000 alone or in combination with PD-1 Ab induces changes in immune markers in the TME, including co-stimulators, markers of cytotoxicity, cytokines and associated receptors, and MHC

**Fig. 3:**
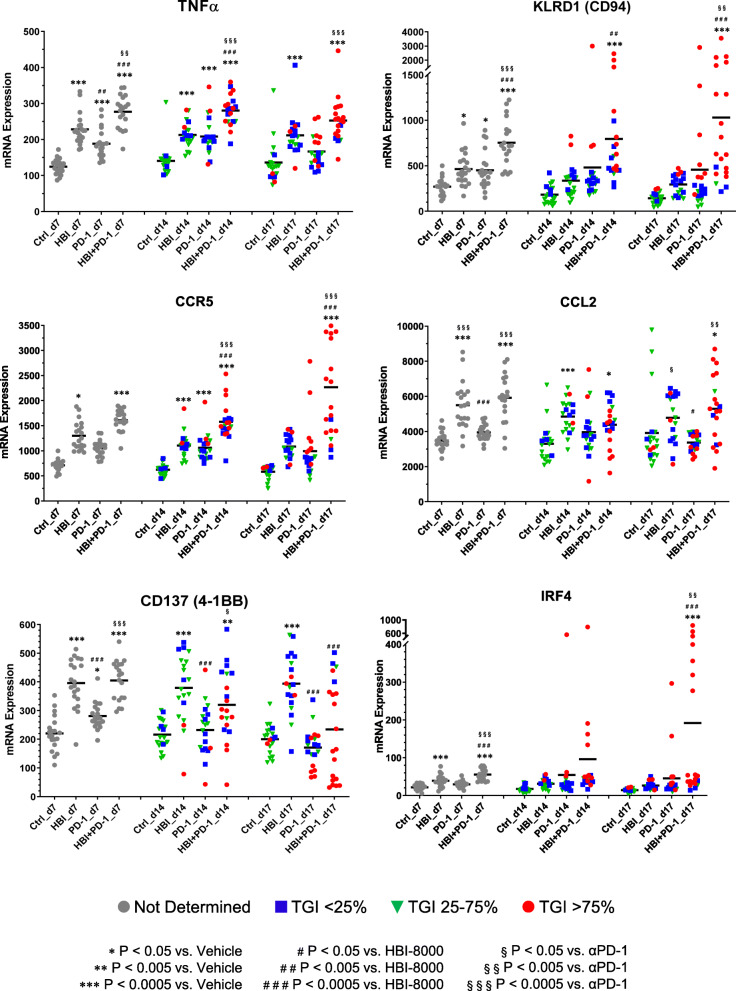
Expression analyses of TNFα, KLRD1, CCR5, CCL2, CD137, and IRF4. Experiments and data analyses as described in Fig. 2

**Fig. 4:**
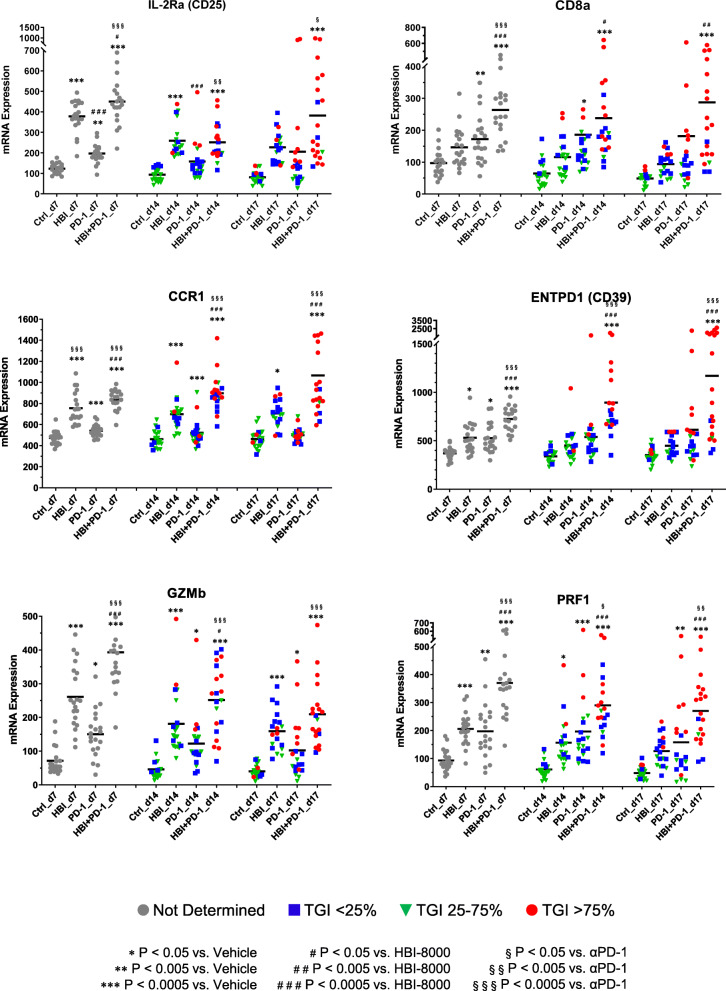
TME immune response-relevant markers modulated by PD-1 Ab, HBI-8000, or their combination. Expression of IL-2Rα, CD8α, CCR1, ENTPD1, GZMB, and PRF1 in tumors isolated from mice in the Vehicle, HBI-8000, PD-1 Ab, and the combination of HBI-8000 and PD-1 Ab groups

**Fig. 5:**
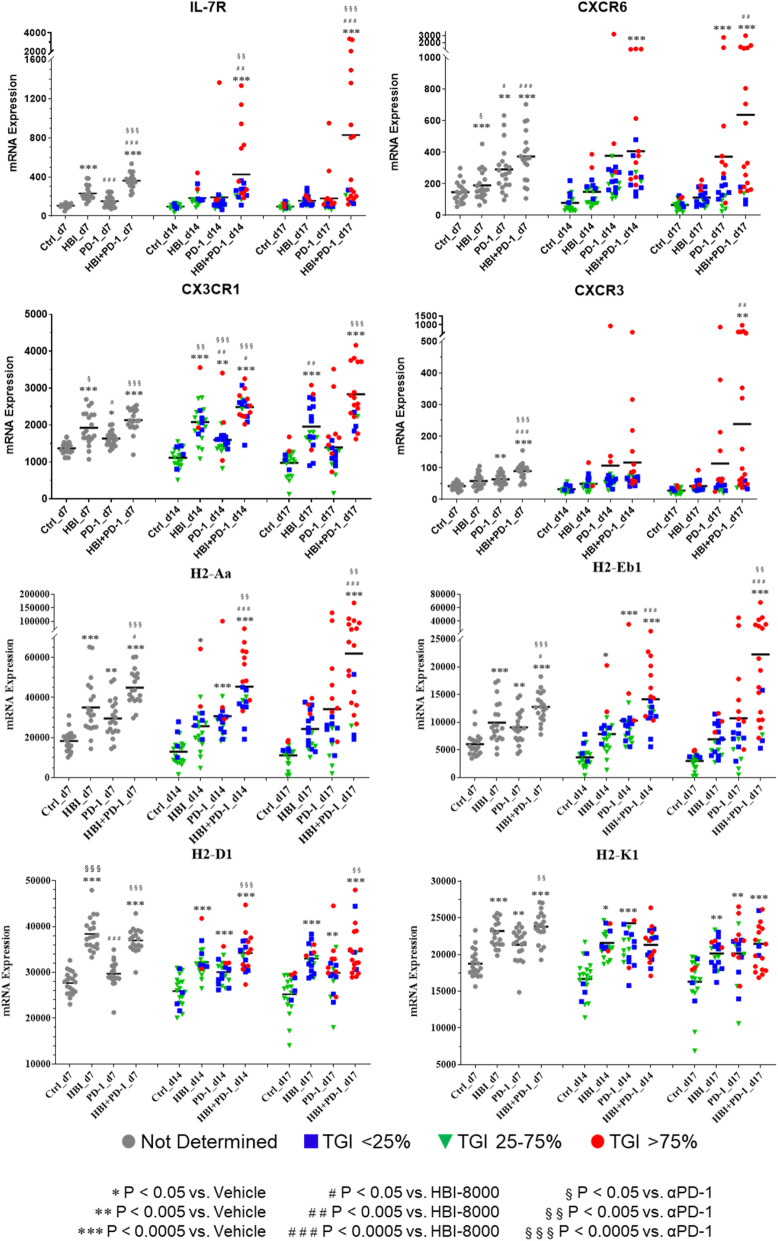
Expression of cytokine/chemokine receptors, MHC class I and class II are modulated by PD-1 Ab, HBI-8000, or their combination. nCounter data analyses (as explained in the Methods and in Fig. 2) identified significant differences in the expression of IL-7R, CXCR6, CX3CR1, CXCR3, H2-Aa, H2-Eb1, H2-D1, and H2-K1 in tumors treated with PD-1 Ab, HBI-8000, or their combination compared to the Vehicle-treated group

In addition to analyzing the effects of HBI-8000, PD-1 Ab, or their combination on various immune pathways, cell type functional scores, and immune checkpoint markers, we examined the effect of HBI-8000, PD-1 Ab, and their combination on a number of individual genes relevant to either innate or adaptive immunity (Figs. 3, 4 and 5, and Supplemental Figures 2 and 3). Genes modulated predominantly by the PD-1 Ab included CD8a (Fig. 4), inducible T cell costimulator (ICOS/CD278), and CD40 (Supplemental Figure 3). PD-1 Ab was also the driver for changes in the expression of genes involved in T cell recruitment, memory, and the CD8 T cell response, including CXCR6 (Fig. 5), ICOS and CD40, (Supplemental Figure 3). Our analysis of the nCounter data (Fig. 4) showed increased T-effector and interferon-γ gene scores, which, along with increases in granzyme B (GZMB) and perforin-1 (PRF1), are collectively consistent with an enhanced T-effector and interferon-γ gene, reflecting enhanced existing immune competency [41, 46–48].

We observed the modulation of many genes affecting the TME inflammation in the tumors treated with the combination of HBI-8000 plus PD-1 Ab. Examples included the co-stimulator CD86 (Fig. 2B), chemoattractant receptors C–C chemokine receptor (CCR) 5 (Fig. 3), and CCR1 (Fig. 4), which are important for initial events in effector T-cell differentiation, markers of increased tumor reactive effector cells, e.g., ectonucleoside triphosphate diphosphohydrolase-1 (ENTPD1/CD39; Fig. 4), PRF1 (Fig. 4), and effector T cell memory precursors (interleukin 7 receptor [IL7R] and interferon regulatory factor 4 [IRF4], Figs. 3 and 5, respectively). Because HBI-8000 enhances both CD8 T cell and NK cell activity and functions (10,30), relevant genes modulated predominantly by HBI-8000 are of great interest. Examples of those genes include: 4-1BB/CD137 (Fig. 3), tumor necrosis factor α (TNFα; Fig. 3), interleukin 2 receptor alpha (IL2Rα)/CD25, GZMB (Fig. 4), IRF4 (Fig. 3), chemokine (C-X3-C motif) receptor 1 (CXC3R1), chemokine (CXC motif) receptor (CXCR)6, and CXCR3 (Fig. 5). These genes are relevant to an initial cytokine or CD8 effector response, tumor infiltrating lymphocyte (TIL) recruitment, effector cell differentiation, and effector memory (31–34).

Importantly, many genes were modulated by HBI-8000 alone relatively early (day 7) in the antitumor response (e.g., CD86, 4-1BB/CD137, TNFα, CCR5, chemokine (C–C motif) ligand 2 (CCL2), IL2Rα/CD25 (Figs. 2b, 3, and 4), and CCR1 and GZMB (Fig. 4). Consistent with reports of HBI-8000 having a positive effect on NK cell functions and innate immunity [26], we observed that HBI-8000 alone or combined with PD-1 Ab modulated the expression of GZMB (Fig. 4), killer cell lectin like receptor D1 (KLRD1/CD94; Fig. 3), and killer cell lectin like receptor C2 (NKG2c/KLRC2), natural killer cell granule protein 7 (NKG7), and killer cell lectin like receptor K1 (KLRK1; Supplemental Figure 3). Finally, and consistent with the upward shifts seen in all scores relevant for antigen presentation machinery and supportive of antigen presentation or tumor cell recognition, we observed increases in the expression of several MHC class I (H2-D1, H2-K1) and II genes (H2-Aa, H2-Eb1) (Fig. 5), in HBI-8000 alone (H2-D1, H2-K1) or the combination of HBI-8000 and PD-1 Ab (H2-Aa, H2-Eb1). This is an important observation and relevant to the reversal of known mechanisms of resistance to ICIs, namely the loss of MHC class I and class II molecules, which impede tumor cell recognition by effector CD8 T cells, as well the presentation of tumor antigens, including neoantigens, to naïve de novo antitumor immune cells [12, 13, 49].

### HBI-8000 combined with ICI rescues mice progressing on single-agent ICI therapy in a model of stable disease leading to acquired resistance and progression

**Fig. 6:**
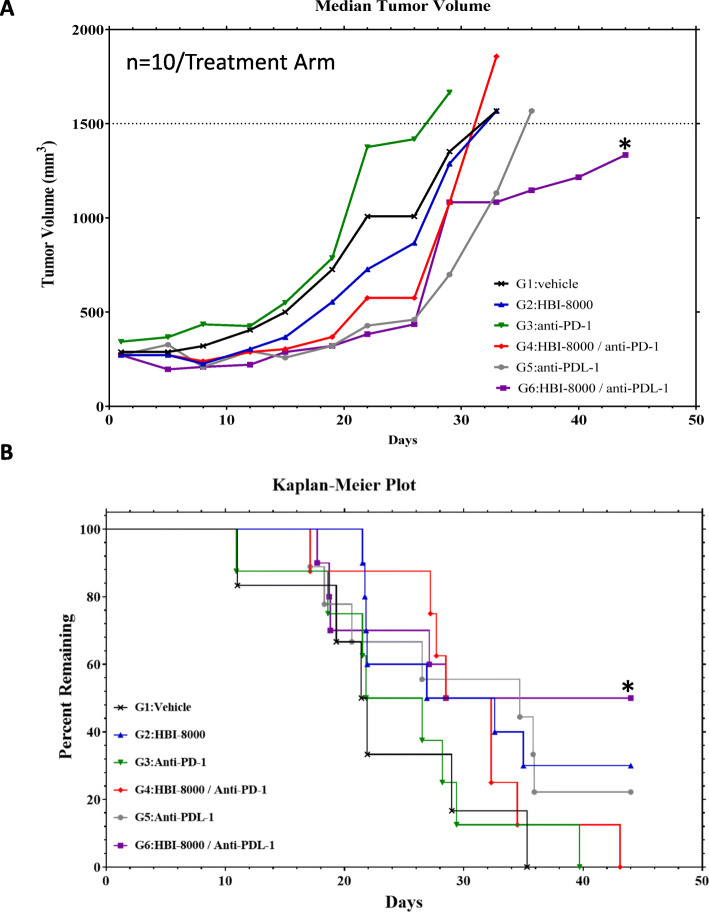
ICI (PD-L1 Ab) plus HBI-8000 reverses resistance to PD-1 Ab therapy and rescues mice with MC38 tumors progressing on PD-1 Ab therapy. Mice implanted with MC38 tumors were treated with PD-1 Ab as a first-line therapy for 18–21 days, at which point mice displaying stable or slow tumor growth were randomized into 1 of 6 s-line treatment groups, including Vehicle, HBI-8000, PD-1 Ab, PD-1 Ab plus HBI-8000, PD-L1 Ab, and PD-L1 Ab plus HBI-8000. Data shown represent median tumor growth (Fig. 6A) and survival (Fig. 6B) in each treatment cohort. * denote statistical significance (*p* < 0.05) when compared HBI-8000 plus anti-PD-L1 vs. anti-PD-1 monotherapy

Human cancer patients receiving ICI therapy often experience a transient response or stable disease, but eventually develop resistance and progress, a challenge to which major efforts are directed. Because gene expression data showed that HBI-8000 alone induced positive changes in a significant number of immune-related pathway scores and genes, we examined the ability of HBI-8000 to halt or even reverse progression in mice first treated with single-agent ICI therapy, alone or in combination with an ICI. To explore the effect of HBI-8000 plus ICI on acquired resistance, we developed a model based on the repeated observations that tumor-bearing mice treated initially (first-line) with single agent PD-1 Ab or PD-L1 Ab display 4 patterns of growth: i) approximately 20% experience rapid progression; ii) approximately 20% experience complete regression, and iii) & iv) approximately 60% experience stable tumor growth (defined as 3 consecutive tumor volume measurements with no significant change) or slow progression (relative to rapid growth and progression), which somewhat approximates the clinical situation. Using the above model, we treated a large cohort of tumor-bearing mice with PD-1 Ab alone. Once they reached the criteria for stable disease or slow progression, they were randomized into 6 treatment arms as indicated in Fig. 6. We compared the effect of halting treatment (Vehicle, Fig. 6A), continuing to treat with PD-1 Ab, or continuing PD-1 Ab in combination with HBI-8000. We also compared the effect of mAbs directed against the reciprocal target, PD-L1, treating mice with PD-L1 Ab, alone or in combination with HBI-8000. As shown in Fig. 6A In mice failing PD-1 Ab therapy, HBI-8000 was modestly efficacious in tumor growth inhibition, however, the second course of PD-1 Ab failed to significantly affect tumor growth. A second course of anti-PD-1 therapy combined with HBI-8000 produced no delay in tumor growth compared with anti-PD-1 alone and the modest delay seen in overall tumor growth provided by treatment with PD-L1 Ab alone was not significant. In contrast, combination therapy with HBI-8000 and anti-PD-L1 significantly (*p* < 0.05) inhibited tumor growth, suggesting that mice progressing on one ICI therapy could see benefit from an alternative ICI in combination with HBI-8000 (Fig. 6A). Analysis of survival, based on terminating mice whose tumors reach 1500 mm^3^, further validated tumor growth curve and suggested a significant delay in tumors regrowth in Group 6 (HBI-8000 plus anti-PD-L1).

## Discussion

Class I-selective HDAC inhibitors reinvigorate the antitumor immune response when combined with ICIs. On the basis of recent reports, we hypothesized that HBI-8000 will function as an epigenetic immunomodulator to reprogram the TME, converting immunologically cold or nonresponsive tumors to hot or responsive tumors, and tested this hypothesis in preclinical syngeneic mouse models of tumor immunotherapy. The ability of HBI-8000 as an HDACi to modulate several immune pathways important to antitumor immunity indicated that these changes in the TME epigenome may significantly improve overall responses to ICIs. This hypothesis is consistent with accumulating evidence that benzamide class I-selective HDACi can reprogram the TME epigenome to improve the antitumor efficacy of ICIs [7, 35–38, 50, 51]. Indeed, HBI-8000 combined with any of the 3 ICIs tested (PD-1 Ab, PD-L1 Ab, and CTLA-4 Ab) displayed enhanced tumor growth inhibition. The nCounter data suggest that the activity of HBI-8000 extended to both adaptive and innate immune functionalities. This is consistent with changes we observed in the expression of several immune checkpoint molecules associated with an immune T cell-inflamed TME. Interestingly, the gene expression responses observed followed 3 patterns (Table 1): i) those that were predominantly driven by PD-1 Ab, ii) those that were predominantly driven by HBI-8000, and iii) those were modulated primarily by the combination, suggesting cooperativity between HBI-8000 and anti-PD-1 in the induction of expression of these genes. Notably, CD276/B7-H3 and CD244/2B4 (Fig. 2B) as well as CD73/NT5E (Supplemental Figure 2) were modulated primarily by HBI-8000, with little or no contribution from the addition of PD-1 Ab, again suggestive of an epigenetic reprograming or “priming” effect on the TME by the HDACi.

**Table 1:**
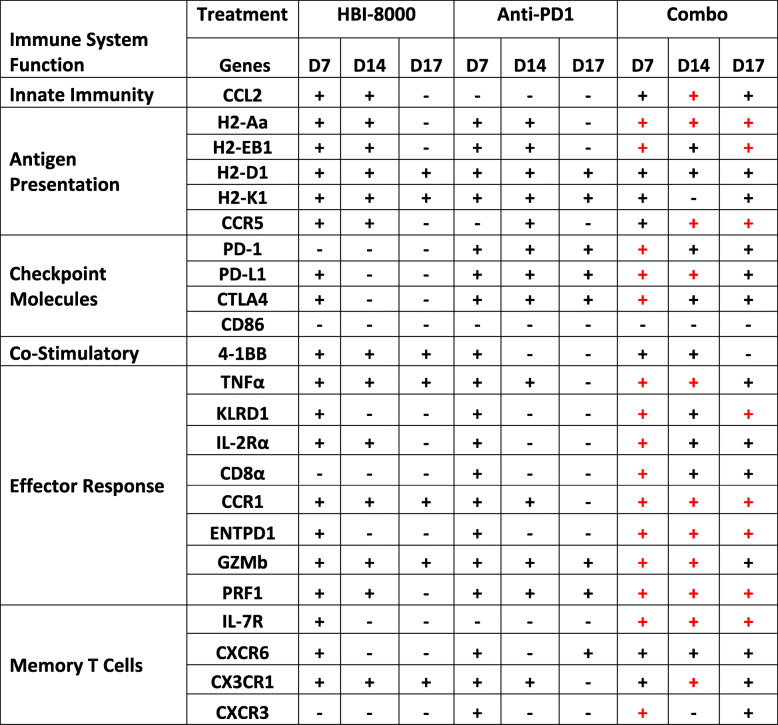
Synergy assessment in differentially expressed genes identified in the nCounter gene expression studies

+ Indicates gene expression is significantly different from that in the vehicle control

(red) indicates a significant difference between the combination treatment and both single-agent treatments

HBI-8000, either alone or in combination with PD-1 Ab, altered the expression of several immune checkpoints, many of which offer potential targets for immunotherapy combinations with HBI-8000. Interestingly, this appeared to be a cooperative effect of HBI-8000 and PD-1 Ab in most cases, as neither agent alone was sufficient. In some cases, however, such as CD276/B7-H3 and CD244/2B4, increased expression was mediated by HBI-8000 alone. CD276 is expressed on antigen-presenting cells and plays an important role in the inhibition of T cell activation and function. The increase in CD276/B7-H3 expression by HBI-8000 may interpret observed augmentation of dendritic cells and associated antigen presenting machinery by HBI-8000. It may also affect the innate immune response and protect tumor cells from NK-mediated cytotoxicity. CD244 is an immunoregulatory receptor in the signaling lymphocyte activation molecule (SLAM) family with both activating and inhibitory properties that seems to function primarily to mediate inhibitory signaling and T cell exhaustion, and offers another potential target for immunotherapy [52].

Tumor-infiltrating lymphocytes are associated with a survival benefit in several cancer types and with the response to immunotherapy [2, 42, 53–58]. The requirements for maintaining a CD8 T cell TIL response against human cancer cells may depend on the presence of stem-like T cells, a distinct subpopulation of CD8 T cells within tumors [59]. Stem-like T cells are delineated by the expression of TCF1, IL7R, and IL2Ra/CD25 (changes observed in our nCounter data) as well as the co-stimulatory molecules CD28, CD226, and CD2. Stem-like T cells terminally differentiate into effector CD8 T cells, which express higher levels of granzymes, perforin, and checkpoint molecules. These stem-like T cells reside in dense antigen-presenting cell niches within the tumor, and tumors that fail to form these structures are not extensively infiltrated by T cells. Moreover, patients with progressive disease lack these immune niches. The increased dendritic cell, MHC class I and II antigen presentation machinery scores together with an increase in both MHC class I and II gene expression driven by HBI-8000 may contribute to the formation and maintenance of these antigen-presenting cell niches, leading to a CD8 T cell TIL response in the TME. Indeed, HBI-8000 in combination with PD-1 Ab or PD-L1 Ab induced an increase in the expression of CD8 in TILs (Fig. 4), along with higher levels of interferon-γ, granzymes, perforin, and checkpoint molecules in treated tumors. It remains unclear if the increase in immune checkpoint activity in the combined regimen with HBI-8000 is a consequence of the epigenetic changes induced directly on tumor or immune cells or the result of a shift in TME cytokine/chemokine profiles [41, 60]. The current data, however, suggest that HBI-8000 alters the TME epigenome, which is necessary for expanding and maintaining both stem-like and effector CD8 cell populations, resulting in more numerous and activated CD8 effector cells as reflected by the increase in the cytotoxic cell, NK CD56dim, CD8 and CD8 vs. exhausted CD8 scores.

An important and under-appreciated mechanism of adaptive tumor resistance is the epigenetic or mutational silencing of the apoptosis machinery. Immunogenic tumor cell death can drive the priming and clonal expansion of tumor-selective effector T cells, but it is ultimately the ability of cytolytic cells to kill tumor cells [61, 62]. HBI-8000 can directly induce cell cycle arrest and apoptosis in a large number of tumor cells and tumor cell lines [26], (data not shown), but has also been shown to potentiate the cytotoxic activity of a number of anticancer agents by skewing the balance of expression toward pro-apoptotic proteins, and thus triggering the apoptotic response [18, 20–32, 63–65]. Based on the current data, as well as recent reports describing immunomodulatory activities of other class I selective HDACi [36, 38, 51, 66], there might be at least 2 mechanisms at play: i) induction of immunomodulatory activities, including boosting antigen presentation and tumor cell recognition by immune effector cells and ii) immunogenic cell death [8, 10, 33, 34, 66–68], leading to the release of neoantigens and a potential increase in T cell priming and de novo generation of new tumor-selective effector T cell clones [69, 70]. Evidence is accumulating that a robust and durable antitumor immune response depends on the generation of novel tumor selective T cell clones [49, 71–73] and not necessarily the reinvigoration or reprogramming of exhausted T cells [11, 74, 75]. The observed shift in the CD8 effector T cell to exhausted T cell ratio may reflect an influx of new tumor-selective T cells.

Using a model of resistance to ICI and tumor progression, we found that second-line HBI-8000 in combination with an ICI rescued a percentage of mice failing ICI therapy (Fig. 6). The ability of HBI-8000 to enable the immune system to target resistant cancer cells may be due in part to its putative effect on antigen presentation and clonal repopulation of the immune response, or its ability to enhance the reinvigoration of exhausted T cells, or both. Ultimately, HBI-8000 and other class I-selective HDACi may epigenetically alter regulatory mechanisms that contribute to achieving a threshold of immunogenic (proinflammatory) signaling that is required to elicit an anti-tumor or autoimmune response [76].

In addition to targeting class I HDACs, HBI-8000 inhibits the activity of class II HDAC10, which is involved in adaptive resistance to the antitumor immune response [77]. In a recent study, knockdown of HDAC10 recapitulated the effects of HDAC inhibitors on immunotherapy biomarkers. Therefore, targeting HDAC10 in addition to inhibiting HDACs 1, 2, and 3 may provide further support for the role of HBI-8000 as an epigenetic modulator and primer of the TME.

In summary, our data may provide a deeper understanding of the effect of class I HDAC inhibitors on the TME. Consistent with the preclinical data presented here, clinical data for HBI-8000 in combination with nivolumab suggest enhancement of activity of nivolumab by HBI-8000 in patients with melanoma, renal cell carcinoma, and non-small cell lung cancer (https://clinicaltrials.gov/ct2/show/NCT02718066), where the durability and sustainability of response appears elevated even after treatment cessation (https://jitc.bmj.com/content/8/Suppl_3/A476.2). This contrasts with other attempts to use HDACi (such as Entinostat) with checkpoint inhibitors to generate clinical responses in patients who have failed prior treatment with ICI (https://www.ascopost.com/News/59894). Differences in safety profile of HDAC inhibitors, sample size, clinical indications and prior treatments with other checkpoint inhibitors might be among factors in determining the outcome of the clinical trials [78]. The current preclinical data may further explain the efficacy and durability of HBI-8000 in combination with nivolumab in the clinical setting. Future studies will be aimed at better understanding the durability of the responses elicited by HBI-8000 by interrogating patient samples through cellular and molecular analysis.

## Supplementary Information

**Additional file 1.**

## Data Availability

The datasets generated and/or analyzed during the current study are available from the corresponding author on reasonable request.

## Abbreviations

Ab: Antibody
CTLA-4: Cytotoxic T-lymphocyte-associated protein 4
CCL2: Chemokine (C-C motif) ligand 2
CCR: Chemokine (C-C motif) receptor
CXCR: Chemokine (CXC motif) receptor
CXC3R1: Chemokine (C-X3-C motif) receptor 1
ENTPD1: Ectonucleoside triphosphate diphosphohydrolase-1
GZMB: Granzyme B
HDAC: Histone deacetylase
HDACi: Histone deacetylase inhibitor
ICIs: Immune checkpoint inhibitors
ICOS: Inducible T cell costimulator
IL2Rα: Interleukin 2 receptor alpha
IL7R: Interleukin 7 receptor
IRF4: Interferon regulatory factor 4
KLRC2: Killer cell lectin like receptor C2
KLRD1: Killer cell lectin like receptor D1
KLRK1: Killer cell lectin like receptor K1
LAG-3: Lymphocyte activation gene-3
mAb: Monoclonal antibody
MHC: Major histocompatibility complex
NK: Natural killer
NKG7: Natural killer cell granule protein 7
NFATC4: Nuclear factor of activated T cells 4
NT5E: Ecto-5′-nucleotidase
PD-1: Programmed cell death receptor-1
PD-L1: Programmed cell death receptor-1 ligand 1
PRF1: Perforin-1
SIRPα: Signal regulatory protein α
TGI: Tumor growth inhibition
TIGIT: T cell immunoreceptor with Ig and ITIM domains
TIL: Tumor infiltrating lymphocyte
TME: Tumor microenvironment
TNFα: Tumor necrosis factor alpha
