# Supplementary material for: The epigenetic immunomodulator, HBI-8000, enhances the response and reverses resistance to checkpoint inhibitors
Source: BMC Cancer. 2021 Aug 30;21:969. doi: 10.1186/s12885-021-08702-x (PMC8404302; doi:10.1186/s12885-021-08702-x)
Supplement: Supplementary file 1 — Additional file 1. [file 12885_2021_8702_MOESM1_ESM.pptx]

## Slide 1
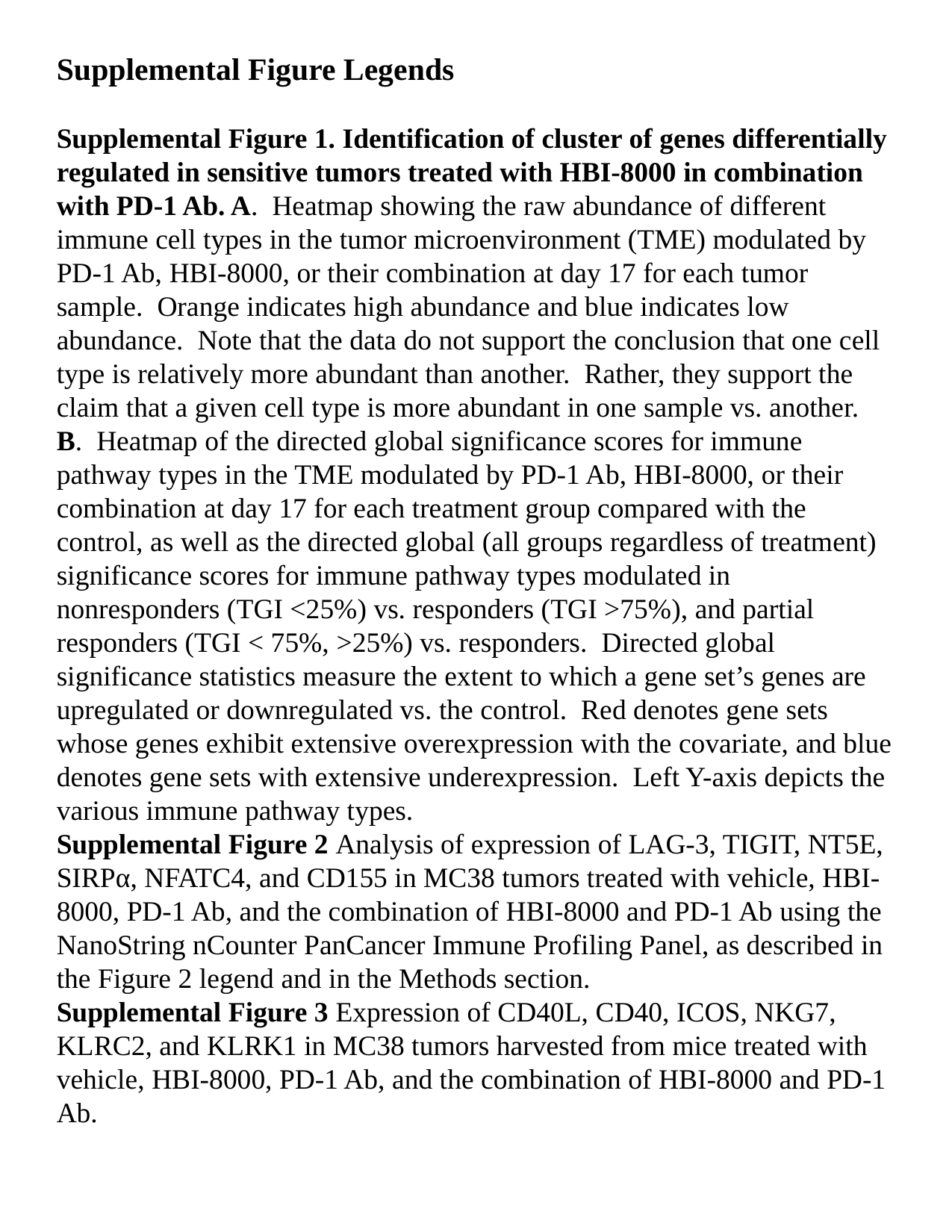

# Supplemental Figure Legends Supplemental Figure 1. Identification of cluster of genes differentially regulated in sensitive tumors treated with HBI-8000 in combination with PD-1 Ab. A. Heatmap showing the raw abundance of different immune cell types in the tumor microenvironment (TME) modulated by PD-1 Ab, HBI-8000, or their combination at day 17 for each tumor sample. Orange indicates high abundance and blue indicates low abundance. Note that the data do not support the conclusion that one cell type is relatively more abundant than another. Rather, they support the claim that a given cell type is more abundant in one sample vs. another. B. Heatmap of the directed global significance scores for immune pathway types in the TME modulated by PD-1 Ab, HBI-8000, or their combination at day 17 for each treatment group compared with the control, as well as the directed global (all groups regardless of treatment) significance scores for immune pathway types modulated in nonresponders (TGI <25%) vs. responders (TGI >75%), and partial responders (TGI < 75%, >25%) vs. responders. Directed global significance statistics measure the extent to which a gene set’s genes are upregulated or downregulated vs. the control. Red denotes gene sets whose genes exhibit extensive overexpression with the covariate, and blue denotes gene sets with extensive underexpression. Left Y-axis depicts the various immune pathway types.   Supplemental Figure 2 Analysis of expression of LAG-3, TIGIT, NT5E, SIRPα, NFATC4, and CD155 in MC38 tumors treated with vehicle, HBI-8000, PD-1 Ab, and the combination of HBI-8000 and PD-1 Ab using the NanoString nCounter PanCancer Immune Profiling Panel, as described in the Figure 2 legend and in the Methods section.  Supplemental Figure 3 Expression of CD40L, CD40, ICOS, NKG7, KLRC2, and KLRK1 in MC38 tumors harvested from mice treated with vehicle, HBI-8000, PD-1 Ab, and the combination of HBI-8000 and PD-1 Ab.

## Slide 2
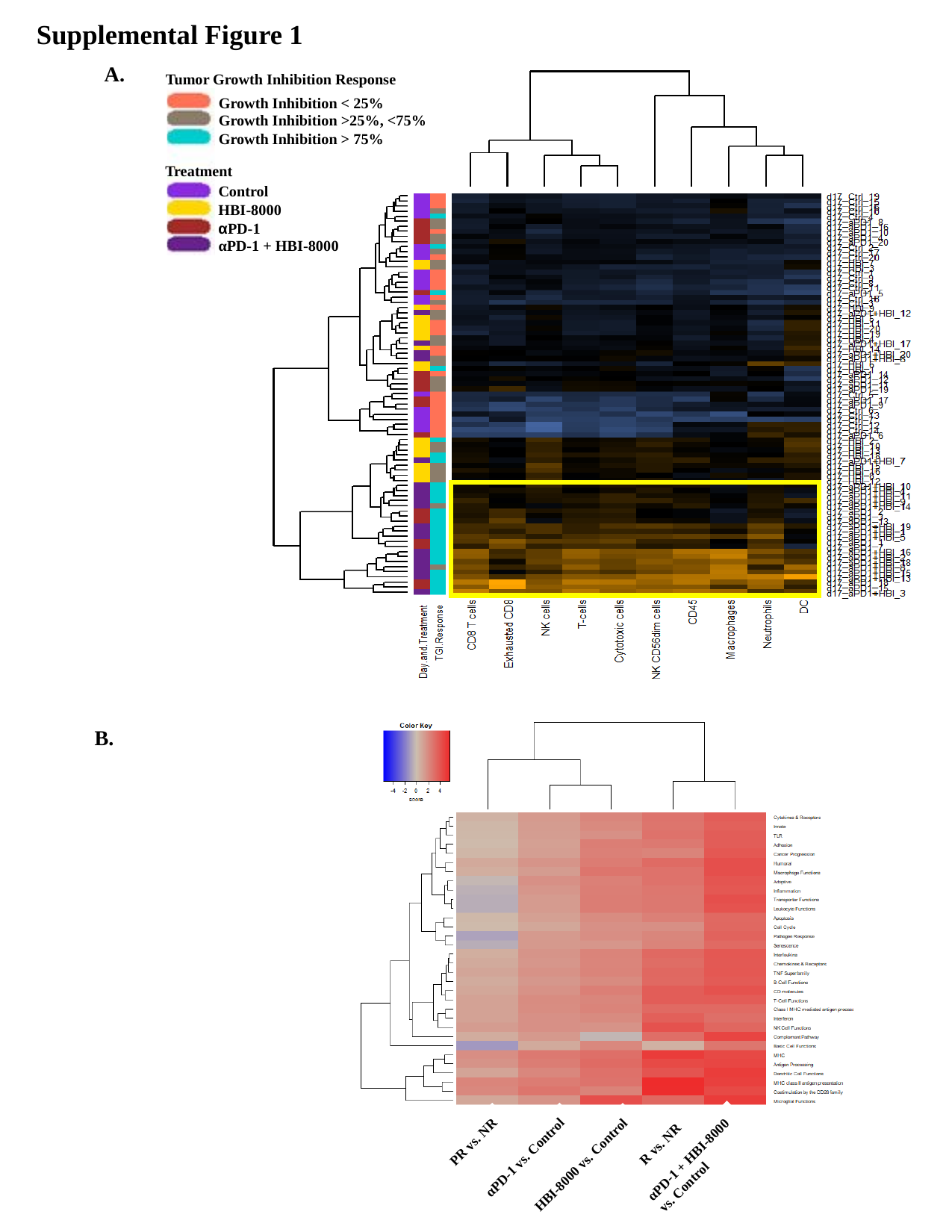

Supplemental Figure 1
A.
Tumor Growth Inhibition Response
Growth Inhibition < 25%
Growth Inhibition >25%, <75%
Growth Inhibition > 75%
Treatment
Control
HBI-8000
αPD-1
αPD-1 + HBI-8000
B.
PR vs. NR
R vs. NR
αPD-1 + HBI-8000
vs. Control
αPD-1 vs. Control
HBI-8000 vs. Control

## Slide 3
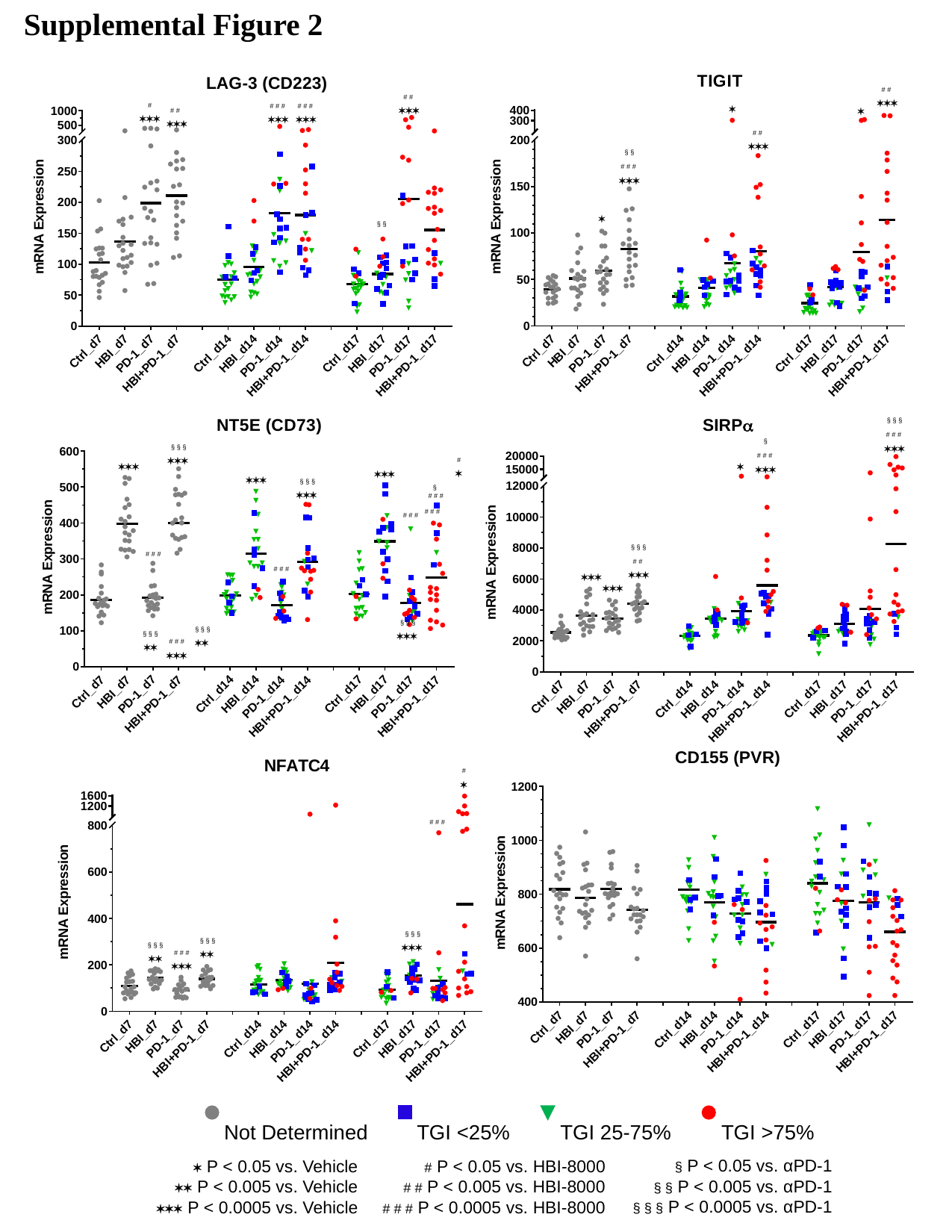

Supplemental Figure 2
# #
 


# #
 
 § §
# # #
 

# #
 
 #
 
# # #
 
# # #
 
# #
 
 § §
 § § §
# # #
 
 §
# # #
 

 § § §
# #
 
 
 
 § § §

 
 
 
 § § §

§
 # # #
 # # #
 # # #
 # # #
#

# # #
 § § §

 § § §

 § § §
 
 # # #
 
#

# # #
 § § §

 § § §

 § § §
 
 # # #
 
Not Determined
TGI <25%
TGI 25-75%
TGI >75%
§ P < 0.05 vs. αPD-1
§ § P < 0.005 vs. αPD-1
§ § § P < 0.0005 vs. αPD-1
 P < 0.05 vs. Vehicle
 P < 0.005 vs. Vehicle
 P < 0.0005 vs. Vehicle
# P < 0.05 vs. HBI-8000
# # P < 0.005 vs. HBI-8000
# # # P < 0.0005 vs. HBI-8000

## Slide 4
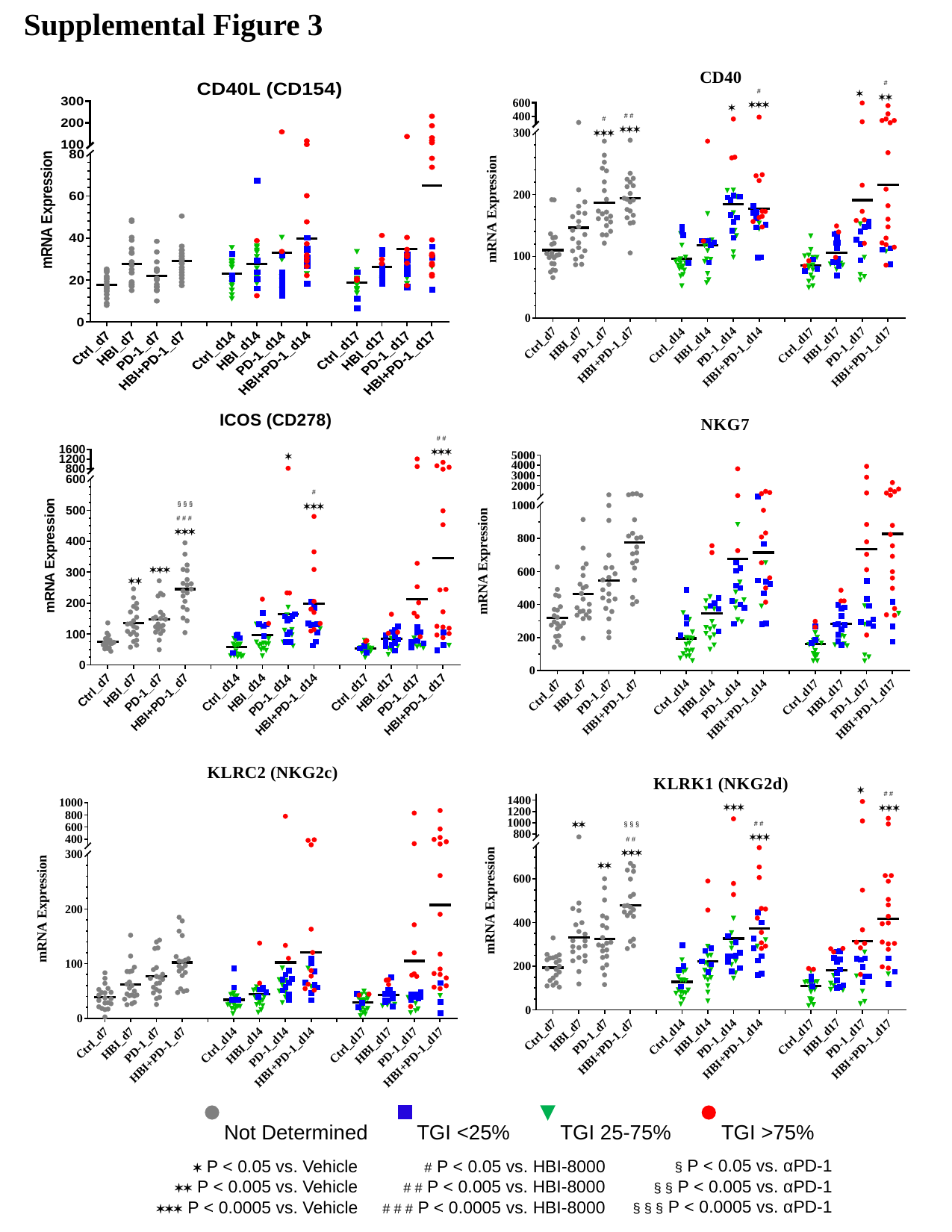

Supplemental Figure 3
 #
 

 #
 

# #
 
 #
 
 # #
 

 #
 
 § § §
# # #
 
 
 

# #
 

# #
 

 § § §
# #
 

Not Determined
TGI <25%
TGI 25-75%
TGI >75%
§ P < 0.05 vs. αPD-1
§ § P < 0.005 vs. αPD-1
§ § § P < 0.0005 vs. αPD-1
 P < 0.05 vs. Vehicle
 P < 0.005 vs. Vehicle
 P < 0.0005 vs. Vehicle
# P < 0.05 vs. HBI-8000
# # P < 0.005 vs. HBI-8000
# # # P < 0.0005 vs. HBI-8000
